# Pneumatosis Intestinalis in the Setting of COVID-19: A Single Center Case Series From New York

**DOI:** 10.3389/fmed.2021.638075

**Published:** 2021-06-04

**Authors:** Santiago J. Miyara, Lance B. Becker, Sara Guevara, Claudia Kirsch, Christine N. Metz, Muhammad Shoaib, Elliot Grodstein, Vinay V. Nair, Nicholas Jandovitz, Alexia McCann-Molmenti, Kei Hayashida, Ryosuke Takegawa, Koichiro Shinozaki, Tsukasa Yagi, Tomoaki Aoki, Mitsuaki Nishikimi, Rishabh C. Choudhary, Young Min Cho, Stavros Zanos, Stefanos Zafeiropoulos, Hannah B. Hoffman, Stacey Watt, Claudio M. Lumermann, Judith Aronsohn, Linda Shore-Lesserson, Ernesto P. Molmenti

**Affiliations:** ^1^Elmezzi Graduate School of Molecular Medicine, Manhasset, NY, United States; ^2^Feinstein Institutes for Medical Research, Manhasset, NY, United States; ^3^Department of Surgery, North Shore University Hospital, Manhasset, NY, United States; ^4^Department of Emergency Medicine, North Shore University Hospital, Manhasset, NY, United States; ^5^Donald and Barbara Zucker School of Medicine at Hofstra/Northwell, Hempstead, NY, United States; ^6^Department of Radiology, North Shore University Hospital, Manhasset, NY, United States; ^7^Department of Medicine, North Shore University Hospital, Manhasset, NY, United States; ^8^Department of Pharmacy, North Shore University Hospital, Manhasset, NY, United States; ^9^Jacobs School of Medicine and Biomedical Sciences, University at Buffalo, Buffalo, NY, United States; ^10^Department of Anesthesiology, North Shore University Hospital, Manhasset, NY, United States

**Keywords:** pneumatosis intestinalis, COVID-19, SARS-CoV-2, mesenteric ischemia, ischemia-reperfusion injury, molecular targeted therapy, tocilizumab, IL-6 inhibitor

## Abstract

This case series reviews four critically ill patients infected with severe acute respiratory syndrome coronavirus 2 (SARS*-*CoV*-*2) [coronavirus disease 2019 (COVID-19)] suffering from pneumatosis intestinalis (PI) during their hospital admission. All patients received the biological agent tocilizumab (TCZ), an interleukin (IL)-6 antagonist, as an experimental treatment for COVID-19 before developing PI. COVID-19 and TCZ have been independently linked to PI risk, yet the cause of this relationship is unknown and under speculation. PI is a rare condition, defined as the presence of gas in the intestinal wall, and although its pathogenesis is poorly understood, intestinal ischemia is one of its causative agents. Based on COVID-19's association with vasculopathic and ischemic insults, and IL-6's protective role in intestinal epithelial ischemia–reperfusion injury, an adverse synergistic association of COVID-19 and TCZ can be proposed in the setting of PI. To our knowledge, this is the first published, single center, case series of pneumatosis intestinalis in COVID-19 patients who received tocilizumab therapy.

## Introduction

Pneumatosis intestinalis (PI) is a rare condition (prevalence ~0.03%) defined as the presence of gas in the wall of the small or large intestine (1–3). PI can represent an incidental, benign finding (primary or idiopathic PI, 15% of cases) or a potentially life-threatening gastrointestinal disease (secondary PI, 85% of cases) (4). Secondary PI is frequently associated with chronic obstructive pulmonary disease (COPD) as well as ischemic, necrotic, and obstructive gastrointestinal insults (5, 6). PI is a radiographic sign characterized by linear and/or curvilinear gas collections in the intestinal wall and is often indicative of systemic or local pathological processes affecting the bowel wall (7, 8). Secondary PI is frequently associated with different clinical scenarios, such as premature newborns with necrotizing enterocolitis, adults with obstructive pulmonary diseases as well as ischemic, necrotic, infectious, and obstructive gastrointestinal insults, celiac disease, amyloidosis, AIDS, rheumatic diseases, and certain drugs, particularly steroids, chemotherapeutics, glucosidase inhibitors, laxatives (lactulose), and molecular targeted agents such as tocilizumab (TCZ) (5, 7, 9–13). The clinical manifestations of PI depend on the bowel segments involved. When PI affects the small intestine, vomiting, abdominal distension, weight loss, and abdominal discomfort/pain are the most common manifestations. Less frequently, diarrhea, anorexia, and constipation can be present. When PI involves the large intestine, diarrhea, hematochezia, abdominal discomfort/pain, and distension are the most common signs and symptoms. Less frequently, constipation, weight loss, and tenesmus may occur (1, 14, 15). The pathogenesis of PI is poorly understood; however, clinical and preclinical studies suggest that PI results from a complex combination of abnormal biochemical, microbiological, and mechanical aspects of intestinal functioning (16).

In December 2019, a novel coronavirus [severe acute respiratory syndrome coronavirus 2 (SARS-CoV-2)] was identified in Wuhan, China as the primary cause of a potentially fatal and multisystemic disease [coronavirus disease 2019 (COVID-19)] (17–19). Since then, three meta-analyses reported that ~15% of COVID-19 patients had GI symptoms, most commonly diarrhea (20–22). GI manifestations in COVID-19 have been acknowledged as early signs of severe/critical disease, usually preceding respiratory symptoms (23, 24). How COVID-19 affects the GI tract is still an open question. Nonetheless, a growing body of evidence suggests that the interaction between SARS-CoV-2 and angiotensin-converting enzyme 2 (ACE2) receptors may result in impaired gut microbiome and immunity (24–26). Furthermore, hypoxia, which putatively has a physiological role in intestinal homeostasis, can be altered as a consequence of COVID-19-induced hypoxia (24, 27).

Systematic reviews and other large observational studies have discussed the association of GI complications in patients undergoing TCZ treatment; however, PI after TCZ use has been recognized by a small number of reports (9, 28, 29). Intestinal perforation is particularly an infrequent but feared complication during TCZ therapy. The exact mechanism of TCZ and GI insults is unknown, but interleukin (IL)-6 antagonism, the primary mechanism of action of TCZ, may impair intestinal homeostasis and recovery capacity after intestinal ischemia (9, 30–33). Remarkably, a history of diverticulitis, non-steroidal anti-inflammatory drugs (NSAIDs), and glucocorticoids use has also been recognized as a risk factor for GI perforation during TCZ treatment (30, 34).

Only four independent case reports have described PI in COVID-19 patients (35–38). Importantly, although TCZ is being utilized as an experimental treatment for COVID-19, the two together may have adverse synergistic effects resulting in increased risk for PI and other related complications. This is the first case series presenting the detailed clinical course of four COVID-19 patients who all received TCZ and developed PI.

## Case Presentation 1

**Figure 1:**
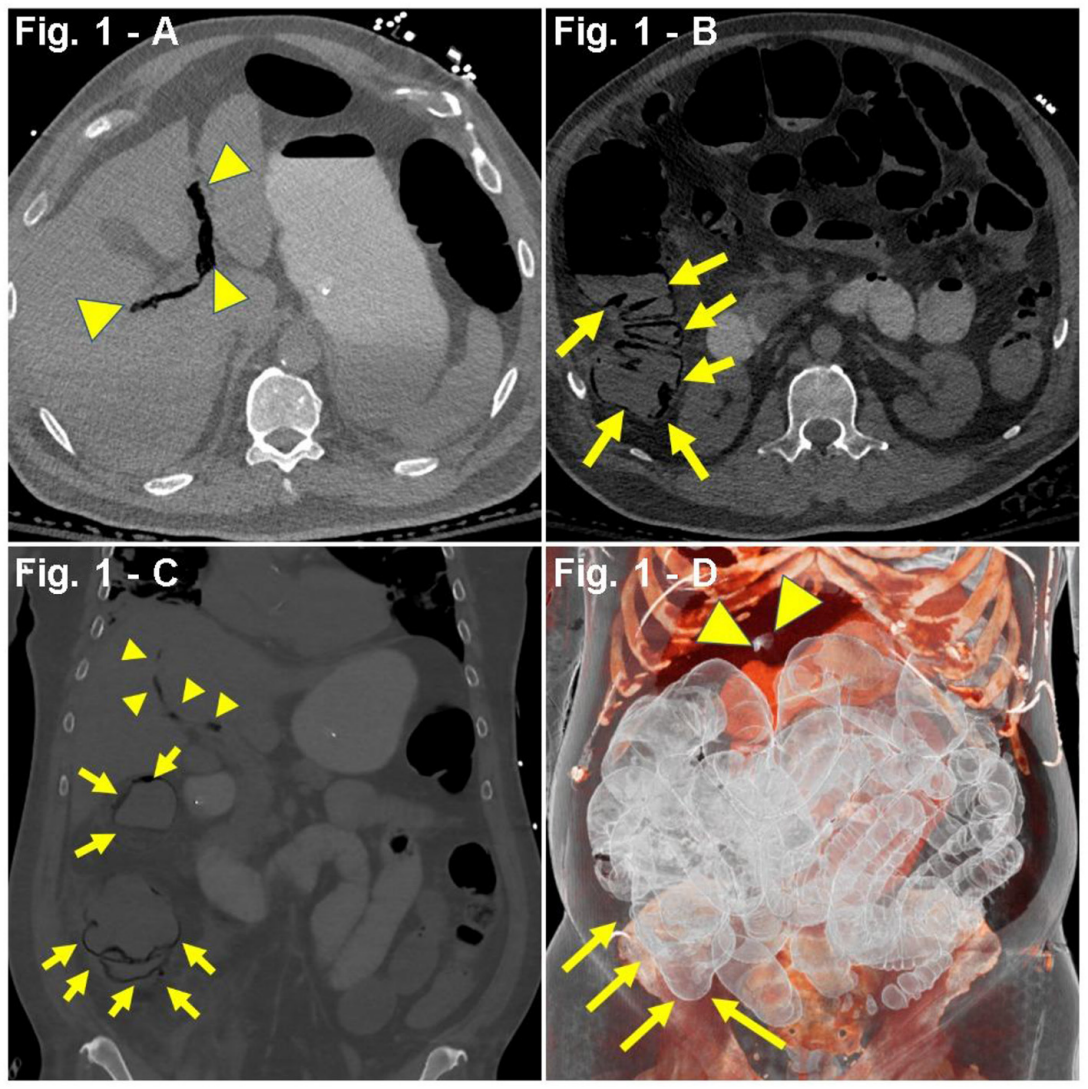
**(A–D)** Case presentation of 65-year-old male patient with COVID-19, 5 days after tocilizumab (TCZ), non-contrast abdominal CT. **(A,B)** Axial, **(C)** coronal, and **(D)** 3D reconstruction, pneumatosis intestinalis (PI) involving ascending colon (yellow arrows), with dilated multiple right lower quadrant small bowel loops with mesenteric and portal venous gas (yellow arrowheads).

A 65-year-old man with a past medical history (PMH) of asthma, hypertension (HTN), hyperlipidemia (HLD), insulin resistance, and obstructive sleep apnea (OSA) was admitted to the hospital due to dyspnea, tachypnea [respiratory rate (RR), 22/min], cough, fever (103°F), and hypoxia [blood oxygen saturation levels (SpO_2_), 81%] after having a 2-week period of fever, chills, and body aches. Symptoms had worsened in the last 72 h before admission. On admission, the patient was placed on a non-rebreather mask (NRB) at 11 L/min (LPM) due to respiratory distress, which partially improved hypoxia from SpO_2_ 81 to 91%. Three days after admission, the respiratory status worsened revealing diffuse bilateral ground-glass opacification on chest CT scan, along with increased counts of neutrophils in plasma, lymphopenia, and transaminitis. Labs showed increased ferritin and C-reactive protein (CRP) levels (Table 1), features compatible with the cytokine release-like syndrome associated with COVID-19 (39, 40). Based on his clinical condition with pending COVID-19 testing results, the patient was started on albuterol, meropenem, hydroxychloroquine, ascorbic acid, thiamine, TCZ, and enoxaparin prophylaxis. One day later, due to decreased oxygen saturation, the patient was placed in pronation. Upon worsening hypoxia, severe dyspnea, positive polymerase chain reaction (PCR) COVID-19 swab testing, and meeting the criteria for acute respiratory distress syndrome (ARDS), the patient was intubated. Furthermore, prophylactic enoxaparin was switched to intravenous (IV) heparin 1,500 U/h, and an IL-1 inhibitor (anakinra) was started. Shortly, a vasoplegic shock refractory to adjustments in sedative medications prompted onset of vasopressors (norepinephrine). Since admission, the patient had no bowel movements despite the use of laxatives (lactulose), prokinetics (metoclopramide), and enemas (saline laxative). Progressive abdominal distension warranted a CT scan demonstrating extensive colon and small bowel pneumatosis with mesenteric and portal venous gas, raising suspicions of bowel ischemia (Figures 1A–D). Based on the patient's clinical and pathological characteristics, lactate levels of 2.0 mmol/L, and no increased vasopressor requirement, surgical resection was not considered the best course of action at the time. Laxative regimen was enhanced with polyethylene glycol and senna. Monitoring of intra-abdominal pressure ranged from 13 to 19 mmHg, which suggested a likely abdominal compartment syndrome (41). Six days after initiation of mechanical ventilation, the patient developed non-oliguric acute kidney injury (AKI) likely secondary to COVID-19 sepsis and acute tubular necrosis (ATN) from hemodynamic instability. The patient was approached in a conservative, non-surgical fashion including switching propofol to ketamine, adjusting IV fluids, using piperacillin–tazobactam for enteric bacteria, and holding potential nephrotoxic agents. A repeated CT scan 12 days after the initial scan showed changes consistent with bowel ischemia, as well as signs suggestive of peritonitis, complicated by bowel perforation, pneumoperitoneum, small bowel obstruction (SBO), enterocutaneous fistulas, and abscess formation. Additionally, the patient developed hematochezia and melena, requiring aggressive resuscitation, including multiple blood and frozen plasma transfusions, as well as repeated drainage procedures to address the intra-abdominal collections. Noteworthy, a CT angiogram ruled out active bleeding at the time of melena and hematochezia. The patient slowly and progressively recovered and was finally discharged for rehabilitation, 90 days after admission. In a delayed fashion, he underwent a right colectomy. Currently, he is alive and well.

**Table 1:** Pneumatosis intestinalis in the setting of COVID-19: case series summary.

	**Past medical history**	**Initial clinical features**	**Abdominal imaging**	**Laboratory results during admission**	**Experimental “COVID-19 drugs” received**	**TCZ administration to PI manifestation (days)**
Case #1	HTN, HLD, ASTHMA, IR, OSA	Dyspnea Tachypnea Hypoxia (SpO_2_, 81%) Cough (non-productive) Fever (103°F) Chills Body aches ARDS	Abd. CT scan: extensive colon and small bowel pneumatosis with mesenteric and portal venous gas (Figures 1A–D).	Neutrophilia (88.8%), lymphopenia (5.2%) Hypokalemia (3.3 mmol/L), transaminitis (AST 64 U/L, ALT 47 U/L) Increased anion gap (18 mmol/L), D-dimer (2,986 ng/mL), CRP (14.17 mg/dL), LDH (545 U/L), and ferritin (1,013 ng/mL) Hypoxia (ABG ~ PaO_2_, 73 mmHg)	Anakinra Ascorbic acid Enoxaparin HCQ TCZ Thiamine	3 days
Case #2	HTN, HLD, DM, OSA	Dyspnea Tachypnea (RR, 40/min) Hypoxia (SpO_2_, 76%) Cough (productive) Fever (103.9°F) Chest pain Chills Myalgias Hyporexia ARDS	Abd. CT scan: air presence in the portal vein and superior mesenteric artery, as well as cecal and small bowel pneumatosis (Figures 2A–D).	Neutrophilia (83%), lymphopenia (11.4%) Increased D-dimer (453 ng/mL), fibrinogen (894 mg/dL), BNP (1,164 pg/mL), CRP (45.34 mg/dL), procalcitonin (0.20 ng/mL), ferritin (1,196 ng/mL), and creatinine (2.01 mg/dL) Hyperglycemia (210 mg/dL), transaminitis (AST; 57 U/L) Hyperlactemia (5.1 mmol/L), hypokalemia (3.2 mEq/L) Oliguria (200 mL/24 h), eGFR (32 mL/min) Hypoxia (ABG~PaO_2_, 51 mmHg) LDH (646 U/L) Metabolic alkalosis (pH 7.48, ${\text{HCO}}_{3}^{-}$ 30 mEq/L, BE 6.2 mmol/L)	AZI CP Enoxaparin MP TCZ	11 days
Case #3	HTN	Dyspnea Cough (non-productive) Hypoxia (SpO_2_, 85%) Fever (100.9°F) Fatigue Non-bloody diarrhea	Abd. CT scan: diffuse small and large bowel pneumatosis (Figures 3A–D).	Neutrophilia (83.9%), lymphopenia (10.5%) Respiratory alkalosis (pH 7.51, ${\text{HCO}}_{3}^{-}$ 24 mEq/L, pCO_2_ 30 mmHg) Hypoxia (ABG ~ PaO_2_, 61 mmHg) Hypoalbuminemia (2.8 g/dL), transaminitis (AST; 161 U/L, ALT; 109 U/L). Increased D-dimer (394 ng/mL), CRP (21.15 mg/dL), LDH (639 U/L), and ferritin (7,378 ng/mL)	Anakinra Enoxaparin HCQ MP TCZ	3 days
Case #4	HTN, DM, Stroke	AMS Upper respiratory symptoms (N/A)	Abd. CT scan: presence of gas in the portal vein and mesenterium as well as extensive bowel pneumatosis (Figures 4A–D).	Leukocytosis (13.76 × 10^9^/L) Increased D-dimer (3,136 ng/mL), procalcitonin (0.70 ng/mL), CRP (3.64 mg/dL), and LDH (982 U/L) Hyperlactemia (8.6 mmol/L), hyperkalemia (5.6 mEq/L) Hyperglycemia (478 mg/dL), hypertriglyceridemia (918 mg/dL) Hypoalbuminemia (3.1 g/dL) Uremia (serum creatinine, 3.85 mg/dL/BUN, 150 mg/dL) eGFR (16 mL/min), oliguria (155 mL/24 h) Mixed acidosis (pH 7.14, ${\text{HCO}}_{3}^{-}$ 18 mEq/L, pCO_2_ 56 mmHg)	HCQ MP Remdesivir TCZ	10 days

*HTN, arterial hypertension; HLD, hyperlipidemia; IR, insulin resistance; OSA, obstructive sleep apnea; DM, diabetes mellitus; CRP, C-reactive protein; HCQ, hydroxychloroquine; TCZ, Tocilizumab; AZI, azithromycin; MP, methylprednisolone; CP, convalescent plasma; AMS, altered mental status; AKI, acute kidney injury; N/A, not available; ARDS, acute respiratory distress syndrome; ABG, arterial blood gases; LDH, lactate dehydrogenase; AST, aspartate aminotransferase; ALT, alanine aminotransferase; BE, base excess; eGFR, estimated glomerular filtration rate*.

## Case Presentation 2

**Figure 2:**
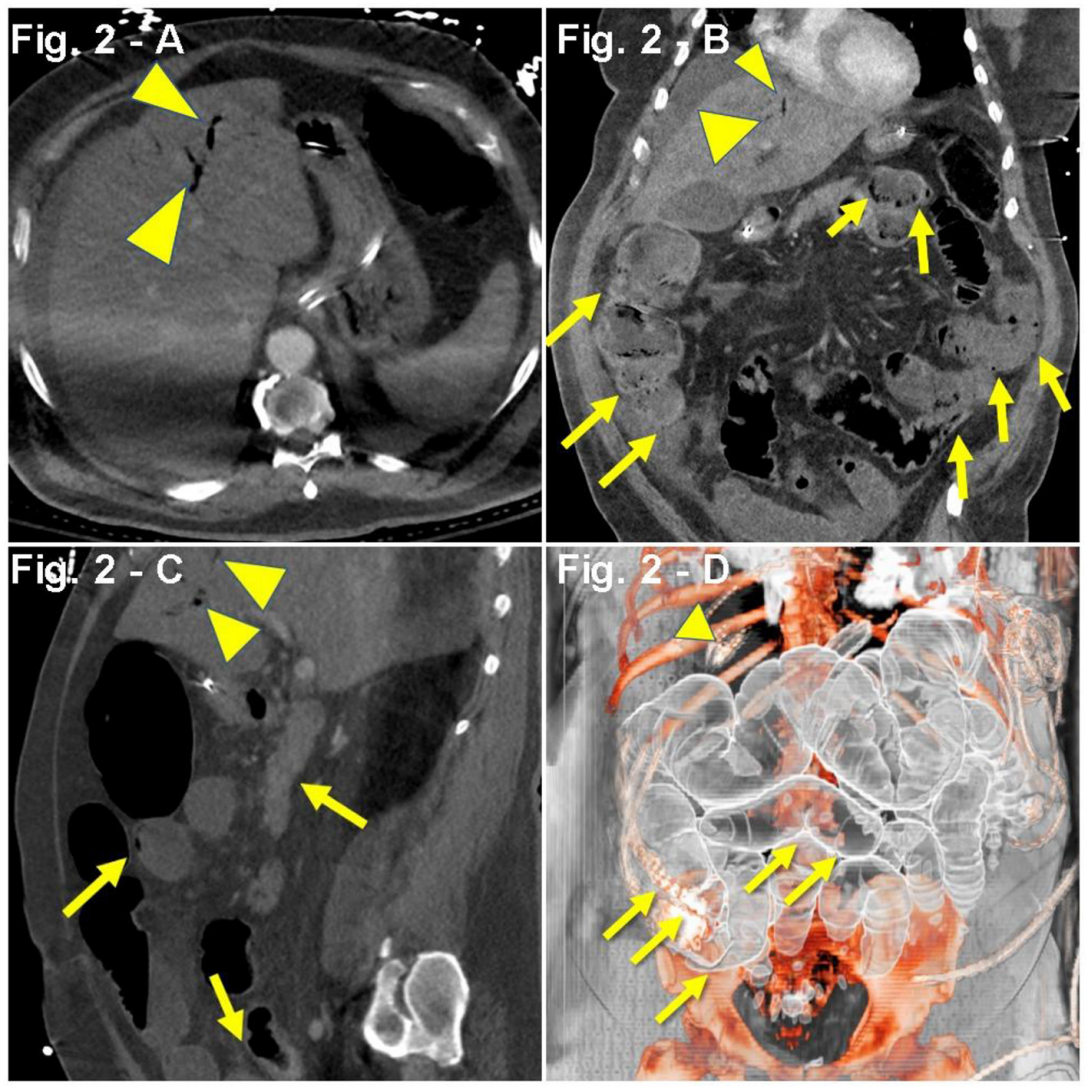
**(A–D)** Case presentation of a 61-year-old male COVID-19 patient with respiratory failure on TCZ with rising lactate, abdominal ileus, abdominal CT with intravenous and oral contrast, **(A)** axial, **(B)** coronal, **(C)** sagittal, and **(D)** 3D reconstruction, with ileus and small and large bowel dilatation, with small bowel and cecal pneumatosis (yellow arrows) with portal gas (yellow arrowheads), and splenic and mesenteric vein gas.

A 61-year-old man with a PMH of HTN, HLD, diabetes mellitus (DM), and OSA was admitted to the hospital after presenting with dyspnea, tachypnea (RR, 40/min), hypoxia (SpO_2_, 76%), worsening cough (productive, non-bloody), fever (103.9°F), chills, myalgias, hyporexia, and chest pain. Symptoms began 4 weeks prior, and 2 weeks prior to admission, he received a course of azithromycin and oseltamivir. One week prior to admission, the patient visited the ED for myalgias, chills, and cough but was discharged with a normal chest X-ray (CXR). The physical examination during the second ED visit was remarkable for severe respiratory distress and bilateral basilar crackles. His hypoxia initially improved with 6 LPM nasal cannula (NC) from SpO_2_ of 76–91%, albeit later requiring 6 L NRB (SpO_2_, 76–95%). CXR revealed bilateral infiltrates, and a CT angiogram with contrast confirmed lung parenchyma compromise with extensive bilateral ground-glass opacities in both lungs. Pulmonary embolism (PE) could not be excluded due to motion associated with the hyperdynamic state. Lab workup revealed positive COVID-19 PCR test through nasal swabbing, lymphopenia, increased D-dimer (453 ng/mL), brain-derived natriuretic peptide (BNP) (1,164 pg/mL), and procalcitonin (0.20 ng/mL) (Table 1). During the first night of admission, the patient became severely hypoxic (PaO_2_, 51%), with improvement in SpO_2_ from 80 to 88% after aggressive resuscitation with steroids (IV methylprednisolone, 100 mg), diuretics (IV furosemide, 40 mg), oxygen (15 LPM NRB), and pronation. On second day of admission, the patient was transferred to the ICU, received one dose of TCZ, and was started on IV methylprednisolone, 50 mg twice daily, IV furosemide, 40 mg daily, inhaled albuterol every 6 h, prophylactic enoxaparin, and non-invasive ventilation (BiLevel 18/14). On the second day of ICU admission, the patient received convalescent plasma (plasma from recovered COVID-19 patients). One week after admission, upon worsening hypoxia and hemodynamic instability, the patient underwent endotracheal intubation for mechanical ventilation and was started on a double regimen of vasopressors (vasopressin, 0.04 U/min and norepinephrine, 0.9 mcg/kg/min). The patient suffered two episodes of arterial thrombosis despite proper anticoagulation with argatroban. Five days after initiating mechanical ventilation, physical examination revealed a protuberant abdomen and dark output from the nasogastric tube (NGT). Further workup showed increased lactate and leukocytosis, hyperkalemia (5.8 mEq/L), increased creatinine (2.01 mg/dL), and oliguria. Abdominal CT scan reported gas in the portal vein and superior mesenteric artery, as well as cecal and small bowel pneumatosis (Figures 2A–D). The benefit of a surgical intervention was considered very low in the setting of an unstable patient with multiorgan failure; hence, it was approached conservatively, including antibiotics (metronidazole and vancomycin), proton-pump inhibitors (pantoprazole), renal replacement therapy [continuous veno-venous hemofiltration (CVVH)], fluid optimization, and metabolic support. On the 10th day of ICU admission, the patient developed refractory cardiopulmonary arrest associated with metabolic acidosis and lactate levels of 24 mmol/L.

## Case Presentation 3

**Figure 3:**
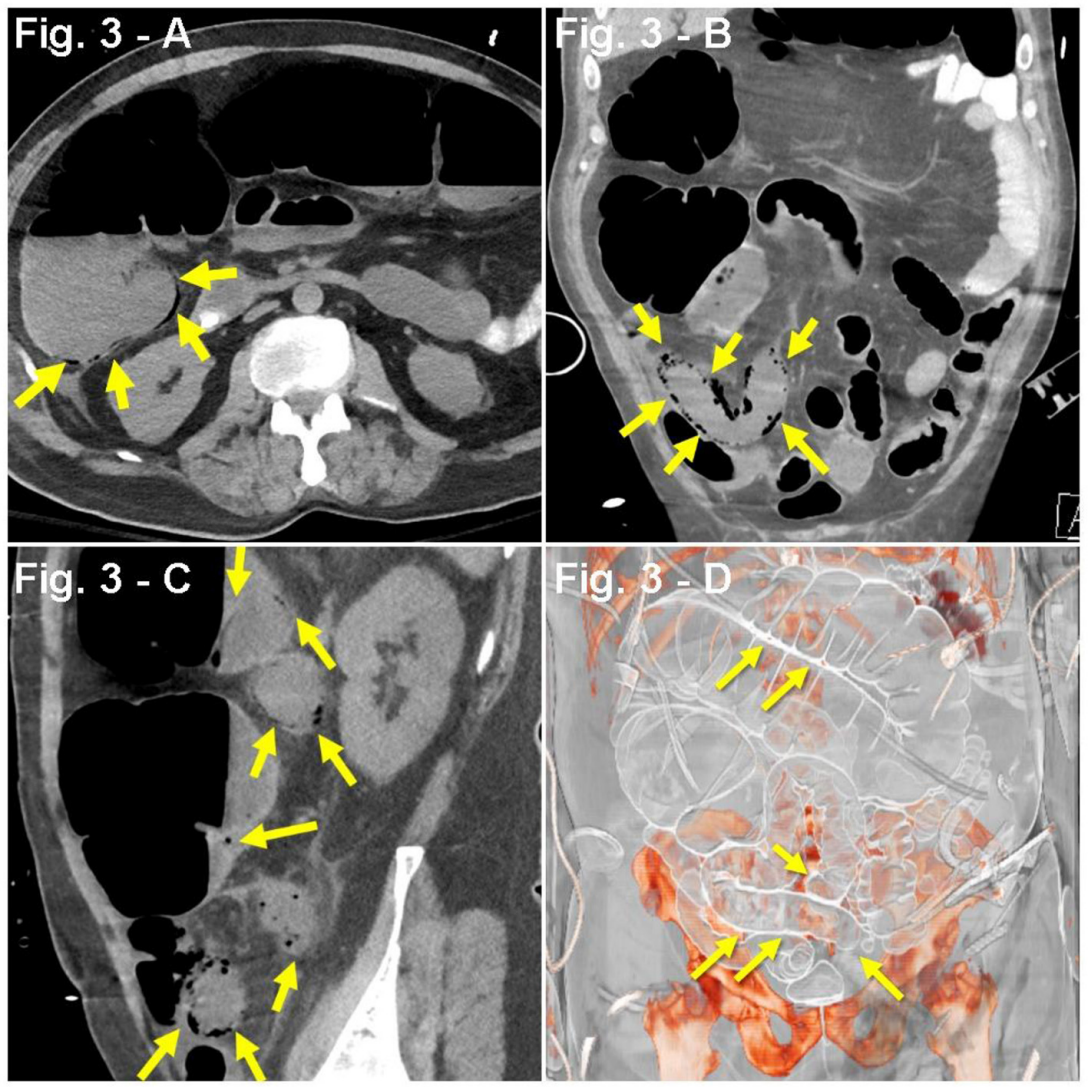
**(A–D)** Case presentation of a 63-year-old male patient, with dyspnea, cough, fever from COVID-19, with bloody diarrhea, and abdominal distention 3 days after receiving TCZ, abdominal CT with oral contrast only, **(A)** axial, **(B)** coronal, **(C)** sagittal, and **(D)** 3D reconstruction with consolidation seen along lung bases, and pneumatosis of small bowel loops (yellow arrows) with dilated small and large bowel loops consistent with ileus.

A 64-year-old man with a PMH of HTN was admitted for 1 week history of dyspnea, cough, fever (100.9°F), and fatigue. Pulse oximetry revealed hypoxia (SpO_2_, 85%), which initially improved to SpO_2_ of 90% with NC at 6 LPM, and later on to SpO_2_ of 92–94% with 10 LPM NRB. A CXR showed bilateral ground-glass opacities. On the second day of admission, the patient developed non-bloody diarrhea. With pending results from PCR COVID-19 nasal swabbing, a presumptive diagnosis of COVID-19 pneumonia was established, and the patient was started on a regimen of PO hydroxychloroquine 200 mg twice daily, IV methylprednisolone 50 mg twice daily, SC prophylactic enoxaparin 40 mg daily, anakinra (IL-1 inhibitor, SC 100 mg every 6 h), and inhaled albuterol. Lab work revealed neutrophilia, lymphopenia, respiratory alkalosis, hypoalbuminemia, transaminitis, as well as increased d-dimer, CRP, lactate dehydrogenase (LDH), and ferritin (Table 1). Despite initial improvement, 5 days after admission, he presented with a nocturnal crisis of hypoxia (SpO_2_, ~60%), which improved after pronation. Four hours later, the patient (without any relevant psychiatric/neurological history) developed intermittent episodes of delirium, agitation, and altered mental status, which was treated with haloperidol. Subsequently, due to worsening hypoxia, the patient underwent sedation, endotracheal intubation, and mechanical ventilation. At this point, the patient was transferred to another ICU and, upon arrival, was found to have an unsecure airway, raising concern for potential aspiration. Based on this, the patient was started on a regimen of IV piperacillin–tazobactam and one dose of vancomycin. Nine days after initial hospital admission, the patient developed septic shock and prerenal acute kidney injury, which prompted hemodynamic support with a norepinephrine drip (0.02 mcg/kg/min). Enoxaparin was switched to IV sodium heparin due to a D-dimer of 987 ng/mL. Additionally, the patient received a single dose of TCZ. Three days after TCZ administration, routine physical examination showed abdominal distension and tympanism with digital percussion. An abdominal X-ray revealed features compatible with colonic ileus or pseudo-obstruction. Subsequent CT scan showed diffuse small and large bowel pneumatosis (Figures 3A–D). This was found in the setting of worsening kidney and liver function, increased ventilation requirements, acidosis (pH 7.17), and leukocytosis (35,000 WBC/mL). Due to broad multiorgan failure, the patient was not deemed a good surgical candidate for segmental resection. CXR showed additional bilateral consolidations in the lower lobes, along with worsening respiratory status, suggesting a superimposed pneumonia. At this point, with the diagnosis of septic shock and multiorgan failure, and considering the ominous prognosis, the family decided to prioritize comfort over other aggressive measures. The patient was withdrawn from mechanical ventilation and developed a cardiopulmonary arrest 4 min thereafter.

## Case Presentation 4

**Figure 4:**
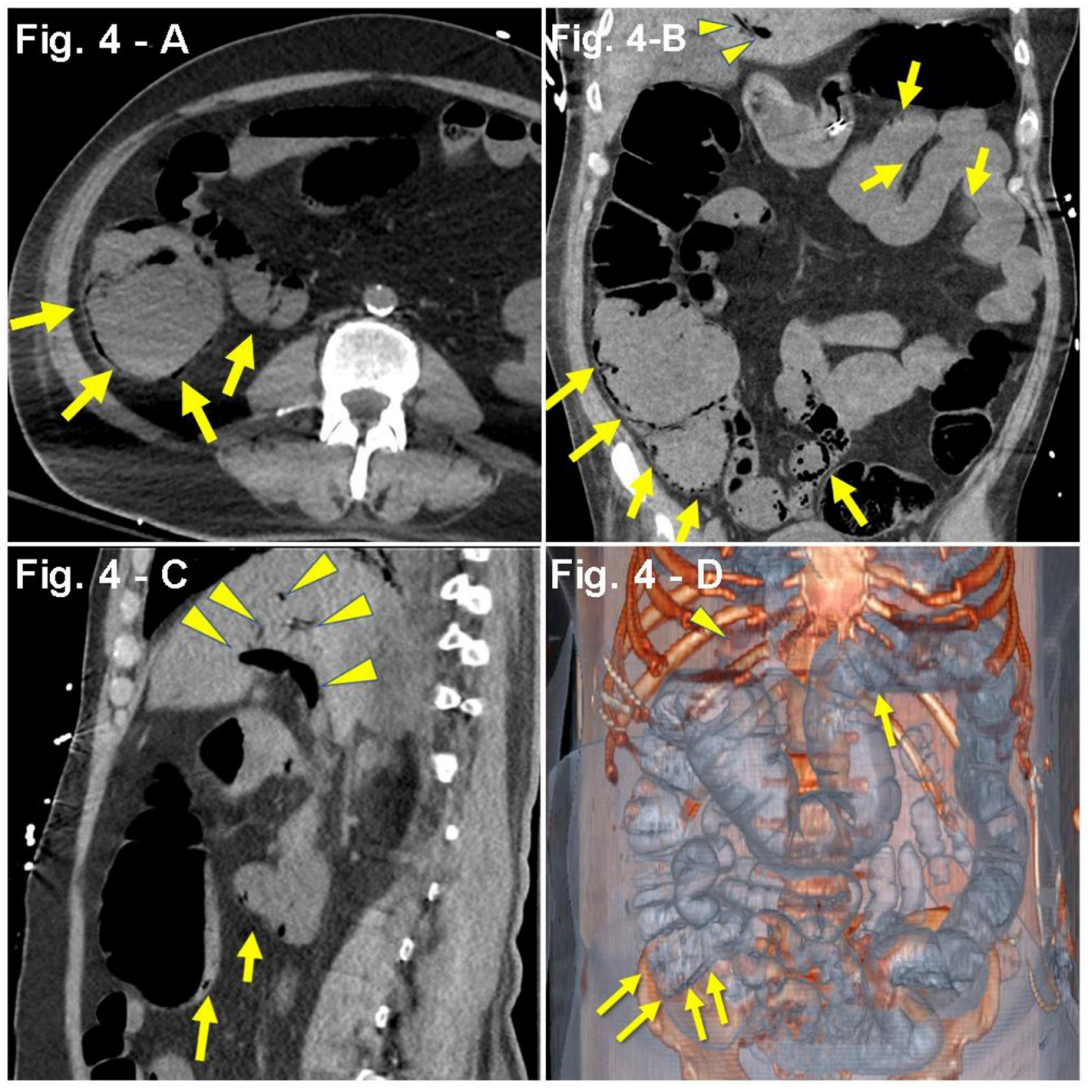
**(A–D)** Case presentation of a 64-year-old male patient, with altered mental status, acute kidney injury, DM2, stroke, bacterial pneumonia with COVID-19, single-dose TCZ, hypotensive, oliguric, non-contrast abdominal CT only, **(A)** axial, **(B)** coronal, **(C)** sagittal, and **(D)** 3D reconstruction with pneumatosis of distal transverse colon, cecum, terminal ileum, and mesenteric venous gas adjacent to the terminal ileum (yellow arrows) concerning for bowel ischemia. There are foci of air in mesenteric vessels in the right lower quadrant, with portal venous gas (yellow arrowheads).

A 64-year-old man with PMH of HTN, insulin-dependent type 2 DM, and stroke 3 years prior with no residual deficits was admitted to an out-of-network hospital due to altered mental status and acute kidney injury (serum creatinine, 2.2 mg/dL). The family reported upper respiratory symptoms 1 week before admission. No acute changes were observed in the patient's head CT scan. Due to concerns of a potential non-ST-segment elevation myocardial infarction (NSTEMI) in the setting of uncontrolled HTN, the patient was started on aspirin, clopidogrel, and a heparin drip. Based on a positive COVID-19 PCR nasal swabbing at admission and concerns for a potential bacterial pneumonia, hydroxychloroquine, remdesivir, ceftriaxone, and doxycycline were initiated. Worsening kidney function parameters (serum creatinine, 2.2–4.2 mg/dL) after antibiotic and antiviral treatment prompted suspension of these medications. One week after admission, the patient received a single dose of TCZ and was started on steroids (IV methylprednisolone, 40 mg every 8 h). By hospital day 10, despite non-invasive ventilation (BiPAP), the progressive worsening of the respiratory status required intubation and mechanical ventilation. One day later, the patient was transferred to our hospital upon family request. On initial assessment, the patient was found to be hypotensive, with oliguria (155 mL/24 h), mixed acidosis (pH 7.04, ${\text{HCO}}_{3}^{-}$ 17 mEq/L, pCO_2_ 56 mmHg), hyperkalemia (5.6 mEq/L), hyperglycemia (478 mg/dL), hypertriglyceridemia (918 mg/dL), hypoalbuminemia (3.1 g/dL), and uremia [serum creatinine 3.85 mg/dL, blood urea nitrogen (BUN) 150 mg/dL], which prompted continuous renal replacement therapy (Table 1). Intensive resuscitation with albumin, bicarbonate, insulin, norepinephrine, and vancomycin was initiated. Propofol was discontinued and switched to dexmedetomidine. The patient also received IV pantoprazole 40 mg, polyethylene glycol, lactulose, senna, and methylnaltrexone. Subsequently, the patient developed fever (101.4°F), leukocytosis (33.16 × 10^9^/L), and low platelets (85 × 10^9^/L). Due to concerns of potential heparin-induced thrombocytopenia, heparin was suspended and replaced with argatroban. Despite intensive supportive care, vasopressor requirements increased prompting the addition of vasopressin at 0.04 U/min. Leukocytes count and lactate further increased (33.16 × 10^9^/L to 40 × 10^9^/L; 2.7–8.6 mmol/L, respectively). One week after the hospital transfer, abdominal distension on physical exam prompted a CT scan that revealed gas in the portal vein and mesentery as well as extensive intestinal pneumatosis (Figures 4A–D). Surgical assessment dismissed a potential bowel resection since the risks were considered greater than any potential benefit. The patient developed refractory septic shock and, 1 day later, a cardiopulmonary arrest.

## Discussion

Pneumatosis intestinalis is thought to result from a complex combination of biochemical, microbiological, and mechanical insults to the intestine (16). The biochemical hypothesis argues that PI originates from excessive hydrogen production by enteric bacteria through chyme fermentation. Support of this hypothesis includes observational studies demonstrating that patients with PI have elevated levels of hydrogen in their breath compared with control patients (42, 43). The microbiological or bacterial hypothesis suggests that gas forming bacteria, such as *Clostridium* species, infringe upon the mucosa through breaches, reaching the submucosa and subsequently forming intramural gas collections (44). Experimental models supporting this hypothesis include the improvement and resolution of the aforementioned intramural gas collections by antibiotic treatment (45). An alternative mechanical hypothesis states that gas can reach the submucosal compartment of the intestinal wall either through breaks in the mucosa (intraluminal source) or the serosal surface (extraluminal source). Indeed, common conditions associated with mucosal disruption are consistently related with PI, such as necrotizing enterocolitis, inflammatory bowel disease, gastrointestinal (GI) tract infections, irritant ingestion, and intestinal ischemia (1). Gas originating extraluminally can be traced to conditions where air can diffuse through tissues, as occurs in COPD (46).

It should be acknowledged that PI is not a disease itself, but rather the imaging manifestation of an underlying pathology or combination of pathologies. As such, the underlying etiology (e.g., ischemia, drugs, infections) must always be addressed accordingly prior to specific treatments (47–49). When PI is accompanied with signs of peritonitis, pH <7.3, ${\text{HCO}}_{3}^{-}$ <20 mEq/L, lactate >2.0 mmol/L, and/or portal venous gas, emergent exploratory laparotomy should be considered (50, 51). Treatment is usually based on the severity of symptoms and can range from repeated imaging in asymptomatic patients to elemental diet for mild symptoms, to antibiotics, oxygen therapy, and hospitalization for severe cases. Notably, clinical judgement is an instrumental component of the decision-making process (7, 45, 52–54).

Since March 11th of 2020, when the global COVID-19 pandemic was formally declared, a growing body of evidence has shown the systemic and extrapulmonary compromise that can potentially occur in the setting of COVID-19 (55). The involvement of neurological, cardiovascular, gastrointestinal, hematopoietic, endocrine, and immune systems have been described in many studies (56–59). Additionally, the ischemic damage seen during COVID-19 infection has also been described in several systematic reviews (60–63). Indeed, observational studies have shown the association of COVID-19 with abdominal ischemia; nonetheless, its pathophysiology remains unknown (64). Research has shown a clear association between high levels of proinflammatory cytokines and inflammatory markers with severe, critical, and fatal forms of COVID-19 (40). Under the logic of specifically targeting the COVID-19-associated “cytokine storm”, trials with specific cytokine inhibitors, such as TCZ, sarilumab, siltuximab (all anti-IL-6), and anakinra (anti-IL-1), have been conducted worldwide^1^. TCZ is an evidence-based treatment for rheumatoid arthritis; however, there are no clear benefits in the setting of COVID-19. At the time of writing, double-blind, randomized, clinical trials have failed to prove any benefit from these drugs in COVID-19 (65, 66). An observational study that included a cohort of 1,351 COVID-19 patients showed that patients receiving TCZ had a decreased risk of invasive mechanical ventilation and death compared to matched controls (67). Another study suggested that TCZ was associated with decreased vasopressor requirements (68). Although PI and gastrointestinal perforation have been recognized as potential adverse effects of TCZ, the mechanisms are completely unknown (9, 30).

Several hypotheses argue in favor of a prothrombotic, microangiopathic, and therefore ischemic effect of COVID-19: (1) SARS-CoV-2 virus can directly invade endothelial cells, resulting in endothelialitis, endothelial dysfunction, and thrombosis (69–72); (2) capillary viscometry showed hyperviscosity in critically ill COVID-19 patients (73); (3) platelet activation and platelet–monocyte aggregation formation in severe COVID-19 patients was documented (74); and (4) thromboelastography (TEG) parameters (decreased R and K values, increased K angle, and MA) consistent with a hypercoagulability state have been found in COVID-19 patients (75).

In short, this case series portrays four critically ill patients who, in the setting of ARDS due to severe COVID-19, received TCZ as an experimental treatment, and all developed complex clinical courses of PI, which subsequently resulted in perforation, sepsis, hemodynamic instability, multiorgan failure, and death in three out of four patients. Herein, we highlight a potential correlation between an infectious disease (COVID-19), an experimental drug in this setting (TCZ), and a rare GI complication (PI). COVID-19 and TCZ have been independently associated with PI (9, 36–38). Intestinal ischemia is a well-established cause of PI; however, the causative mechanism of PI during TCZ treatment is unknown. Preclinical studies suggested that IL-6 plays a critical role in intestinal epithelial proliferation and repair after ischemia–reperfusion, traumatic, and microbiological insults (30, 76). Clinical studies have shown IL-6's pivotal role in vascular endothelial growth factor production, as well as angiogenesis and wound healing (31–33).

Taken together, we venture to think that a possible negative and devastating synergy occurs between the microvascular insults from COVID-19, along with the lack of epithelial protection and vascular support from IL-6 blocking, ultimately resulting in intestinal wall damage, epithelium dysfunction, and intraluminal gas diffusion (Pneumatosis Intestinalis). However, it must be acknowledged that COVID-19 may affect intestinal homeostasis by other complex mechanisms involving gut microbiome and barrier functioning (24). To the best of our knowledge, this is the first case series of PI in COVID-19 patients, as well as the first reported from the same institution, and the first report of COVID-19 patients treated with TCZ who subsequently developed PI.

At the time of writing, COVID-19 is a novel entity with poorly understood pathology. Experimental, emergency, and compassionate use of drugs in COVID-19 has been the object of recent discussions. It should always be cautioned that deleterious interactions between drug-related adverse effects and intrinsic features of an infectious disease, in this case COVID-19, can lead to further complications (77, 78) “*Primum non nocere*”.

## Limitations

Discussions regarding the causal relationship between COVID-19 and TCZ with PI are beyond the scope of this publication. However, it is important to acknowledge that some medications that patients received (methylprednisolone and lactulose) may be linked to PI development (1, 13, 79–81). Furthermore, these four critically-ill patients received intensive care support, which inherently adds numerous variables, including known and unknown interactions between each other and the host, resulting in an uncertain number of potential confounders. Additionally, two patients (Cases 3 and 4) received initial care at an outside hospital, and although the hospital course summary was accessible, the variability of clinical setting may hide unknown confounders. Therefore, it is complex and not possible for the authors to establish any definite cause–effect connection between the described variables (COVID-19 and TCZ) with the highlighted clinical outcome (PI). Further preclinical and clinical research addressing interactions between COVID-19 and TCZ with PI is warranted.

## Data Availability Statement

The original contributions presented in the study are included in the article/supplementary material, further inquiries can be directed to the corresponding author.

## Ethics Statement

The studies involving human participants were reviewed and approved by Study approved by IRB #: 20-0884, Feinstein Institutes for Medical Research. Written informed consent for participation was not required for this study in accordance with the national legislation and the institutional requirements. Written informed consent was not obtained from the individual(s) for the publication of any potentially identifiable images or data included in this article.

## Author Contributions

SM, LB, SG, CK, KH, and EM prepared the manuscript's first draft. SM, LB, and EM retrieved and corroborated the data. CK prepared the figures and edited the manuscript. LS-L, JA, TA, KS, EG, VN, NJ, CM, TY, RT, YC, MS, RC, SW, HH, MN, and SZaf collaborated in the discussion. AM-M, SZan, and CL collaborated in the review. All authors contributed to manuscript revision, read, and approved the submitted version.

## Conflict of Interest

The authors declare that the research was conducted in the absence of any commercial or financial relationships that could be construed as a potential conflict of interest.

## Footnotes

^1^Available online at: https://clinicaltrials.gov/ct2/results?cond=&term=tocilizumab+covid-19&cntry=&state=&city=&dist=.

## References

[1] Pear BL. Pneumatosis intestinalis: a review. Radiology. (1998) 207:13–9. 10.1148/radiology.207.1.9530294 9530294

[2] Boerner RM Fried DB Warshauer DM Isaacs K. Pneumatosis intestinalis. Digest Dis Sci. (1996) 41:2272–85. 10.1007/BF02071412 8943984

[3] Kang G. Benign pneumatosis intestinalis: dilemma for primary care clinicians. Can Fam Phys. (2017) 63:766–8. 29025802 PMC5638473

[4] Saul T Palamidessi N. Pneumatosis intestinalis. J Emerg Med. (2011) 40:545–6. 10.1016/j.jemermed.2008.10.019 19272737

[5] Doumit M Saloojee N Seppala R. Pneumatosis intestinalis in a patient with chronic bronchiectasis. Can J Gastroenterol. (2008) 22:595297. 10.1155/2008/595297 18925310 PMC2661306

[6] Iida A Naito H Tsukahara K Yumoto T Nosaka N Kawana S. Pneumatosis cystoides intestinalis presenting as pneumoperitoneum in a patient with chronic obstructive pulmonary disease: a case report. J Med Case Rep. (2017) 11:1–3. 10.1186/s13256-017-1198-2 28241852 PMC5329944

[7] Heng Y Schuffler MD Haggitt RC Rohrmann CA. Pneumatosis intestinalis: a review. Am J Gastroenterol. (1995) 90:1747–58. 7572888

[8] Khalil PN Huber-Wagner S Ladurner R Kleespies A Siebeck M Mutschler W. Natural history, clinical pattern, and surgical considerations of pneumatosis intestinalis. Euro J Med Res. (2009) 14:231–9. 10.1186/2047-783X-14-6-231 19541582 PMC3352014

[9] Jacobs B Jawad A Fattah Z. Pneumatosis intestinalis and intestinal perforation in a patient receiving tocilizumab. Arch Rheumatol. (2018) 33:372. 10.5606/ArchRheumatol.2018.6668 30632527 PMC6328221

[10] Dawe N Akhtar S. Pneumatosis intestinalis presenting with a pneumoperitoneum in a patient with chronic bronchiectasis: a delayed diagnosis of superior mesenteric artery ischaemia. BMJ Case Rep. (2010) 2010:bcr0120102622. 10.1136/bcr.01.2010.2622 22767661 PMC3027758

[11] Dhadlie S Mehanna D McCourtney J. Pneumatosis intestinalis a trap for the unwary: case series and literature review. Int J Surg Case Rep. (2018) 53:214–7. 10.1016/j.ijscr.2018.10.079 30428434 PMC6232619

[12] Ezuka A Kawana K Nagase H Takahashi H Nakajima A. Improvement of pneumatosis cystoides intestinalis after steroid tapering in a patient with bronchial asthma: a case report. J Med Case Rep. (2013) 7:163. 10.1186/1752-1947-7-163 23803391 PMC3704706

[13] Goodman RA Riley TR 3rd. Lactulose-induced pneumatosis intestinalis and pneumoperitoneum. Dig Dis Sci. (2001) 46:2549–53. 10.1023/a:1012308911096 11713968

[14] Wu LL Yang YS Dou Y Liu QS. A systematic analysis of pneumatosis cystoids intestinalis. World J Gastroenterol. (2013) 19:4973. 10.3748/wjg.v19.i30.4973 23946603 PMC3740428

[15] Jamart J. Pneumatosis cystoides intestinalis. A statistical study of 919 cases. Acta Hepato Gastroenterol. (1979) 26:419–22. 525221

[16] Blair HA Baker R Albazaz R. Pneumatosis intestinalis an increasingly common radiological finding, benign or life-threatening? A case series. Case Rep. (2015) 2015:bcr2014207234. 10.1136/bcr-2014-207234 25694632 PMC4336884

[17] World Health Organization. WHO Director-General's Remarks at the Media Briefing on 2019-nCoV on 11 February 2020. Geneva: World Health Organization. Available online at: https://www.who.int/dg/speeches/detail/who-director-general-s-remarks-at-the-media-briefing-on-2019-ncov-on-11-february-2020 (accessed April 10, 2020).

[18] Temgoua MN Endomba FT Nkeck JR Kenfack GU Tochie JN Essouma M. Coronavirus disease 2019 (COVID-19) as a multi-systemic disease and its impact in low- and middle-income countries (LMICs). SN Compr Clin Med. (2020). 10.1007/s42399-020-00417-7. [Epub ahead of print]. 32838173 PMC7371790

[19] World Health Organization. Pneumonia of Unknown Cause—China. Available online at: https://www.who.int/csr/don/05-january-2020-17pneumonia-of-unknown-cause-china/en/ (accessed January 5, 2020).

[20] Cheung KS Hung IF Chan PP Lung KC Tso E Liu R. Gastrointestinal manifestations of SARS-CoV-2 infection and virus load in fecal samples from the Hong Kong cohort and systematic review and meta-analysis. Gastroenterology. (2020) 159:81–95. 10.1053/j.gastro.2020.03.065 32251668 PMC7194936

[21] Sultan S Altayar O Siddique SM Davitkov P Feuerstein JD Lim JK. AGA Institute rapid review of the gastrointestinal and liver manifestations of COVID-19, meta-analysis of international data, and recommendations for the consultative management of patients with COVID-19. Gastroenterology. (2020) 159:320–34. 10.1053/j.gastro.2020.05.001 32407808 PMC7212965

[22] Mao R Qiu Y He JS Tan JY Li XH Liang J. Manifestations and prognosis of gastrointestinal and liver involvement in patients with COVID-19: a systematic review and meta-analysis. Lancet Gastroenterol Hepatol. (2020) 5:667–78. 10.1016/S2468-1253(20)30126-6 32405603 PMC7217643

[23] Jin X Lian JS Hu JH Gao J Zheng L Zhang YM. Epidemiological, clinical and virological characteristics of 74 cases of coronavirus-infected disease 2019 (COVID-19) with gastrointestinal symptoms. Gut. (2020) 69:1002–9. 10.1136/gutjnl-2020-320926 32213556 PMC7133387

[24] Trottein F Sokol H. Potential causes and consequences of gastrointestinal disorders during a SARS-CoV-2 infection. Cell Rep. (2020) 32:107915. 10.1016/j.celrep.2020.107915 32649864 PMC7332457

[25] Liang W Feng Z Rao S Xiao C Xue X Lin Z. Diarrhoea may be underestimated: a missing link in 2019 novel coronavirus. Gut. (2020) 69:1141–3. 10.1136/gutjnl-2020-320832 32102928

[26] Gu S Chen Y Wu Z Chen Y Gao H Lv L. Alterations of the gut microbiota in patients with COVID-19 or H1N1 influenza. Clin Infect Dis. (2020) 71:2669–78. 10.1093/cid/ciaa709 32497191 PMC7314193

[27] Singhal R Shah YM. Oxygen battle in the gut: hypoxia and hypoxia-inducible factors in metabolic and inflammatory responses in the intestine. J Biol Chem. (2020) 295:10493–505. 10.1074/jbc.REV120.011188 32503843 PMC7383395

[28] Gout T Östör AJ Nisar MK. Lower gastrointestinal perforation in rheumatoid arthritis patients treated with conventional DMARDs or tocilizumab: a systematic literature review. Clin Rheumatol. (2011) 30:1471. 10.1007/s10067-011-1827-x 21833686

[29] Strangfeld A Richter A Siegmund B Herzer P Rockwitz K Demary W. Risk for lower intestinal perforations in patients with rheumatoid arthritis treated with tocilizumab in comparison to treatment with other biologic or conventional synthetic DMARDs. Ann Rheumatic Dis. (2017) 76:504–10. 10.1136/annrheumdis-2016-209773 27405509 PMC5445993

[30] Kuhn KA Manieri NA Liu TC Stappenbeck TS. IL-6 stimulates intestinal epithelial proliferation and repair after injury. PLoS ONE. (2014) 9:e114195. 10.1371/journal.pone.0114195 25478789 PMC4257684

[31] Nakahara H Song J Sugimoto M Hagihara K Kishimoto T Yoshizaki K. Anti–interleukin-6 receptor antibody therapy reduces vascular endothelial growth factor production in rheumatoid arthritis. Arthritis Rheumatism. (2003) 48:1521–9. 10.1002/art.11143 12794819

[32] Verheul HM Pinedo HM. Possible molecular mechanisms involved in the toxicity of angiogenesis inhibition. Nat Rev Cancer. (2007) 7:475–85. 10.1038/nrc2152 17522716

[33] Pawar A Desai RJ Solomon DH Ortiz AJ Gale S Bao M. Risk of serious infections in tocilizumab versus other biologic drugs in patients with rheumatoid arthritis: a multidatabase cohort study. Ann Rheumatic Dis. (2019) 78:456–64. 10.1136/annrheumdis-2018-214367 30679153

[34] Malaviya AP Ledingham J Bloxham J Bosworth A Buch M Choy E. The 2013 BSR and BHPR guideline for the use of intravenous tocilizumab in the treatment of adult patients with rheumatoid arthritis. Rheumatology. (2014) 53:1344–6. 10.1093/rheumatology/keu168 24821853

[35] Aiello P Johnson S Mercado AR Hussein S. Pneumatosis intestinalis in a patient with COVID-19. BMJ Case Reports CP. (2020) 13:e237564. 10.1136/bcr-2020-237564 PMC7478032 32900750

[36] Meini S Zini C Passaleva MT Frullini A Fusco F Carpi R. Pneumatosis intestinalis in COVID-19. BMJ Open Gastroenterol. (2020) 7:e000434. 10.1136/bmjgast-2020-000434 PMC7287500 32522754

[37] Lakshmanan S Toubia N. Pneumatosis intestinalis in COVID-19. Clin Gastroenterol Hepatol. (2020) 7:e000434. 10.1016/j.cgh.2020.05.048 PMC7260506 32485300

[38] Kielty J Duggan WP O'Dwyer M. Extensive pneumatosis intestinalis and portal venous gas mimicking mesenteric ischaemia in a patient with SARS-CoV-2. Ann R Coll Surg Engl. (2020) 102:e145–7. 10.1308/rcsann.2020.0145 32538098 PMC7388941

[39] Moore JB June CH. Cytokine release syndrome in severe COVID-19. Science. (2020) 368:473–4. 10.1126/science.abb8925 32303591

[40] Mehta P McAuley DF Brown M Sanchez E Tattersall RS Manson JJ. COVID-19: consider cytokine storm syndromes and immunosuppression. Lancet. (2020) 395:1033. 10.1016/S0140-6736(20)30628-0 32192578 PMC7270045

[41] De Laet IE Malbrain ML De Waele JJ. A clinician's guide to management of intra-abdominal hypertension and abdominal compartment syndrome in critically ill patients. Crit Care. (2020) 24:1–9. 10.1186/s13054-020-2782-1 32204721 PMC7092484

[42] Read NW Al-Janabi MN Cann PA. Is raised breath hydrogen related to the pathogenesis of pneumatosis coli?. Gut. (1984) 25:839–45. 10.1136/gut.25.8.839 6745722 PMC1432562

[43] Christl SU Gibson GR Murgatroyd PR Scheppach W Cummings JH. Impaired hydrogen metabolism in pneumatosis cystoides intestinalis. Gastroenterology. (1993) 104:392–7. 10.1016/0016-5085(93)90406-3 8425681

[44] Yale CE Balish E Wu JP. The bacterial etiology of pneumatosis cystoides intestinalis. Arch Surg. (1974) 109:89–94. 10.1001/archsurg.1974.01360010067017 4365449

[45] Ellis BW. Symptomatic treatment of primary pneumatosis coli with metronidazole. Br Med J. (1980) 280:763. 10.1136/bmj.280.6216.763-a 7370646 PMC1600585

[46] Keyting WS McCarver RR Kovarik JL Daywitt AL. Pneumatosis intestinalis: a new concept. Radiology. (1961) 76:733–41. 10.1148/76.5.733 13752845

[47] Shinagare AB Howard SA Krajewski KM Zukotynski KA Jagannathan JP Ramaiya NH. Pneumatosis intestinalis and bowel perforation associated with molecular targeted therapy: an emerging problem and the role of radiologists in its management. Am J Roentgenol. (2012) 199:1259–65. 10.2214/AJR.12.8782 23169717

[48] Poor A Braman SS. Pneumatosis intestinalis associated with the tyrosine kinase inhibitor nintedanib. Lung. (2018) 196:373–5. 10.1007/s00408-018-0118-6 29721603

[49] Yanaru R Hizawa K Nakamura S Yoshimura R Watanabe K Nakamura U. Regression of pneumatosis cystoides intestinalis after discontinuing of alpha-glucosidase inhibitor administration. J Clin Gastroenterol. (2002) 35:204–5. 10.1097/00004836-200208000-00020 12172373

[50] Knechtle SJ Davidoff AM Rice RP. Pneumatosis intestinalis. Surgical management and clinical outcome. Ann Surg. (1990) 212:160. 10.1097/00000658-199008000-00008 2375647 PMC1358051

[51] Höer J Truong S Virnich N Füzesi L Schumpelick V. Pneumatosis cystoides intestinalis: confirmation of diagnosis by endoscopic puncture a review of pathogenesis, associated disease and therapy and a new theory of cyst formation. Endoscopy. (1998) 30:793–9. 10.1055/s-2007-1001424 9932761

[52] Holt S Gilmour HM Buist TA Marwick K Heading RC. High flow oxygen therapy for pneumatosis coli. Gut. (1979) 20:493–8. 10.1136/gut.20.6.493 468075 PMC1412444

[53] Grieve DA Unsworth IP. Pneumatosis cystoides intestinalis: an experience with hyperbaric oxygen treatment. Aust N Zeal J Surg. (1991) 61:423–6. 10.1111/j.1445-2197.1991.tb00255.x 2059174

[54] Zhang H Jun SL Brennan TV. Pneumatosis intestinalis: not always a surgical indication. Case Rep Surg. (2012) 2012:719713. 10.1155/2012/719713 23198249 PMC3502823

[55] Ng OT Marimuthu K Chia PY Koh V Chiew CJ De Wang L. SARS-CoV-2 infection among travelers returning from Wuhan, China. N Engl J Med. (2020) 382:1476–8. 10.1056/NEJMc2003100 32163698 PMC7121487

[56] Duarte-Neto AN Monteiro RAA da Silva LFF Malheiros DMAC de Oliveira EP Theodoro-Filho J. Pulmonary and systemic involvement in COVID-19 patients assessed with ultrasound-guided minimally invasive autopsy. Histopathology. (2020) 77:186–97. 10.1111/his.14160 32443177 PMC7280721

[57] Driggin E Madhavan MV Bikdeli B Chuich T Laracy J Biondi-Zoccai G. Cardiovascular considerations for patients, health care workers, and health systems during the COVID-19 pandemic. J Am Coll Cardiol. (2020) 75:2352–71. 10.1016/j.jacc.2020.03.031 32201335 PMC7198856

[58] Muller I Cannavaro D Dazzi D Covelli D Mantovani G Muscatello A. SARS-CoV-2-related atypical thyroiditis. Lancet Diabetes Endocrinol. (2020) 8:739–41. 10.1016/S2213-8587(20)30266-7 32738929 PMC7392564

[59] Bangash MN Patel J Parekh D. COVID-19 and the liver: little cause for concern. Lancet Gastroenterol Hepatol. (2020) 5:529. 10.1016/S2468-1253(20)30084-4 32203680 PMC7270582

[60] Tan YK Goh C Leow AS Tambyah PA Ang A Yap ES. COVID-19 and ischemic stroke: a systematic review and meta-summary of the literature. J Thromb Thromb. (2020) 50:587–95. 10.1007/s11239-020-02228-y 32661757 PMC7358286

[61] Merkler AE Parikh NS Mir S Gupta A Kamel H Lin E. Risk of ischemic stroke in patients with coronavirus disease 2019 (COVID-19) vs patients with influenza. JAMA Neurol. (2020) 77:1–7. 10.1001/jamaneurol.2020.2730 32614385 PMC7333175

[62] Mao L Jin H Wang M Hu Y Chen S He Q. Neurologic manifestations of hospitalized patients with coronavirus disease 2019 in Wuhan, China. JAMA Neurol. (2020) 77:683–90. 10.1001/jamaneurol.2020.1127 32275288 PMC7149362

[63] Helms J Kremer S Merdji H Clere-Jehl R Schenck M Kummerlen C. Neurologic features in severe SARS-CoV-2 infection. N Engl J Med. (2020) 382:2268–70. 10.1056/NEJMc2008597 32294339 PMC7179967

[64] Bhayana R Som A Li MD Carey DE Anderson MA Blake MA. Abdominal imaging findings in COVID-19: preliminary observations. Radiology. (2020) 297:E207–15. 10.1148/radiol.2020201908 32391742 PMC7508000

[65] Strand V Burmester GR Ogale S Devenport J John A Emery P. Improvements in health-related quality of life after treatment with tocilizumab in patients with rheumatoid arthritis refractory to tumour necrosis factor inhibitors: results from the 24-week randomized controlled RADIATE study. Rheumatology. (2012) 51:1860–9. 10.1093/rheumatology/kes131 22753773 PMC3448882

[66] Roche. Roche provides an update on the phase III COVACTA trial of Actemra/RoActemra in hospitalised patients with severe COVID-19 associated pneumonia. Available online at: https://www.roche.com/dam/jcr:6d8de90d-2e31-43c8-b4e1-0a24a2675015/en/29072020-mr-covacta.pdf

[67] Guaraldi G Meschiari M Cozzi-Lepri A Milic J Tonelli R Menozzi M. Tocilizumab in patients with severe COVID-19: a retrospective cohort study. Lancet Rheumatol. (2020) 2:e474–84. 10.1016/S2665-9913(20)30173-9 32835257 PMC7314456

[68] Kewan T Covut F Al–Jaghbeer MJ Rose L Gopalakrishna KV Akbik B. Tocilizumab for treatment of patients with severe COVID−19: a retrospective cohort study. EClinicalMedicine. (2020) 24:100418. 10.1016/j.eclinm.2020.100418 32766537 PMC7305505

[69] Ackermann M Verleden SE Kuehnel M Haverich A Welte T Laenger F. Pulmonary vascular endothelialitis, thrombosis, and angiogenesis in Covid-19. N Engl J Med. (2020) 382:120–8. 10.1056/NEJMoa2015432 32437596 PMC7412750

[70] Teuwen LA Geldhof V Pasut A Carmeliet P. COVID-19: the vasculature unleashed. Nat Rev Immunol. (2020) 20:1–3. 10.1038/s41577-020-0356-8 32439870 PMC7240244

[71] Libby P Lüscher T. COVID-19 is, in the end, an endothelial disease. Euro Heart J. (2020) 41:3038–44. 10.1093/eurheartj/ehaa623 PMC7470753 32882706

[72] Varga Z Flammer AJ Steiger P Haberecker M Andermatt R Zinkernagel AS. Endothelial cell infection and endotheliitis in COVID-19. Lancet. (2020) 395:1417–8. 10.1016/S0140-6736(20)30937-5 32325026 PMC7172722

[73] Maier CL Truong AD Auld SC Polly DM Tanksley CL Duncan A. COVID-19-associated hyperviscosity: a link between inflammation and thrombophilia?. Lancet. (2020) 395:1758–9. 10.2139/ssrn.3598209 32464112 PMC7247793

[74] Hottz ED Azevedo-Quintanilha IG Palhinha L Teixeira L Barreto EA Pão CR. Platelet activation and platelet-monocyte aggregate formation trigger tissue factor expression in patients with severe COVID-19. Blood J Am Soc Hematol. (2020) 136:1330–41. 10.1182/blood.2020007252 32678428 PMC7483437

[75] Panigada M Bottino N Tagliabue P Grasselli G Novembrino C Chantarangkul V. Hypercoagulability of COVID-19 patients in intensive care unit. A report of thromboelastography findings and other parameters of hemostasis. J Thromb Haemost. (2020) 18:1738–42. 10.1111/jth.14850 32302438 PMC9906150

[76] Jin X Zimmers TA Zhang Z Pierce RH Koniaris LG. Interleukin-6 is an important *in vivo* inhibitor of intestinal epithelial cell death in mice. Gut. (2010) 59:186–96. 10.1136/gut.2008.151175 19074180

[77] Jiang S. Don't rush to deploy COVID-19 vaccines and drugs without sufficient safety guarantees. Nature. (2020) 579:321. 10.1038/d41586-020-00751-9 32179860

[78] Grein J Ohmagari N Shin D Diaz G Asperges E Castagna A. Compassionate use of remdesivir for patients with severe Covid-19. N Engl J Med. (2020) 382:2327–36. 10.1056/NEJMoa2007016 32275812 PMC7169476

[79] Ohara H Kato Y Nakano M Ishii Y Serizawa H Watanabe N. A case of pneumatosis cystoides intestinalis induced by steroid pulse therapy for severe acute hepatitis B. Nihon Shokakibyo Gakkai Zasshi. (2011) 108:1237. 10.11405/nisshoshi.108.1237 21737976

[80] Varelas LJ Klinge MJ Malik SM Borhani AA Neal M. Idiopathic Pneumatosis intestinalis secondary to lactulose use in patients with cirrhosis. J Gastroenterol Hepatol. (2020) 35:1065–8. 10.1111/jgh.14920 31692099

[81] Lee CI Wu YH. Pneumatosis intestinalis and pneumoretroperitoneum post steroid use in a patient with superior mesenteric artery syndrome. Am J Emerg Med. (2019) 37:1993-e1–3. 10.1016/j.ajem.2019.06.040 31262624

